# Distinct and Contrasting Transcription Initiation Patterns at *Mycobacterium tuberculosis* Promoters

**DOI:** 10.1371/journal.pone.0043900

**Published:** 2012-09-07

**Authors:** Priyanka Tare, Arnab China, Valakunja Nagaraja

**Affiliations:** 1 Department of Microbiology and Cell Biology, Indian Institute of Science, Bangalore, India; 2 Jawaharlal Nehru Centre for Advanced Scientific Research, Bangalore, India; University of Delhi, India

## Abstract

Although sequencing of *Mycobacterium tuberculosis* genome lead to better understanding of transcription units and gene functions, interactions occurring during transcription initiation between RNA polymerase and promoters is yet to be elucidated. Different stages of transcription initiation include promoter specific binding of RNAP, isomerization, abortive initiation and promoter clearance. We have now analyzed these events with four promoters of *M. tuberculosis viz* ***.*** P*_gyrB1_*, P*_gyrR_*, P*_rrnPCL1_* and P*_metU_*. The promoters differed from each other in their rates of open complex formation, decay, promoter clearance and abortive transcription. The equilibrium binding and kinetic studies of various steps revealed distinct rate limiting events for each of the promoter, which also differed markedly in their characteristics from the respective promoters of *Mycobacterium smegmatis*. Surprisingly, the transcription at *gyr* promoter was enhanced in the presence of initiating nucleotides and decreased in the presence of alarmone, pppGpp, a pattern typically seen with rRNA promoters studied so far. The *gyr* promoter of *M. smegmatis*, on the other hand, was not subjected to pppGpp mediated regulation. The marked differences in the transcription initiation pathway seen with *rrn* and *gyr* promoters of *M. smegmatis* and *M. tuberculosis* suggest that such species specific differences in the regulation of expression of the crucial housekeeping genes could be one of the key determinants contributing to the differences in growth rate and lifestyle of the two organisms. Moreover, the distinct rate limiting steps during transcription initiation of each one of the promoters studied point at variations in their intracellular regulation.

## Introduction

*M. tuberculosis* is one of the most formidable pathogens known to mankind. The resurgence of the pathogen, its alliance with HIV infection and emergence of the drug resistant strains has resulted in a global challenge to combat tuberculosis [1]. The distinctive features of the bacterium such as slow growth rate, dormancy, unique cell wall composition, resistance towards phagocytosis by macrophages etc demand a thorough investigation of its biology at the molecular level. A number of studies carried out so far reveal significant differences in the transcription process in mycobacteria when compared to *E. coli* and other bacteria [2], [3]. Presence of as many as 13 sigma factors for transcription from promoters with diverse architecture [2], [4], [5] and inability of the mycobacterial promoters to function in *E. coli* [5]–[7] are some of the key features warranting a detailed study of the transcription process in the pathogen.

**Figure 1:**
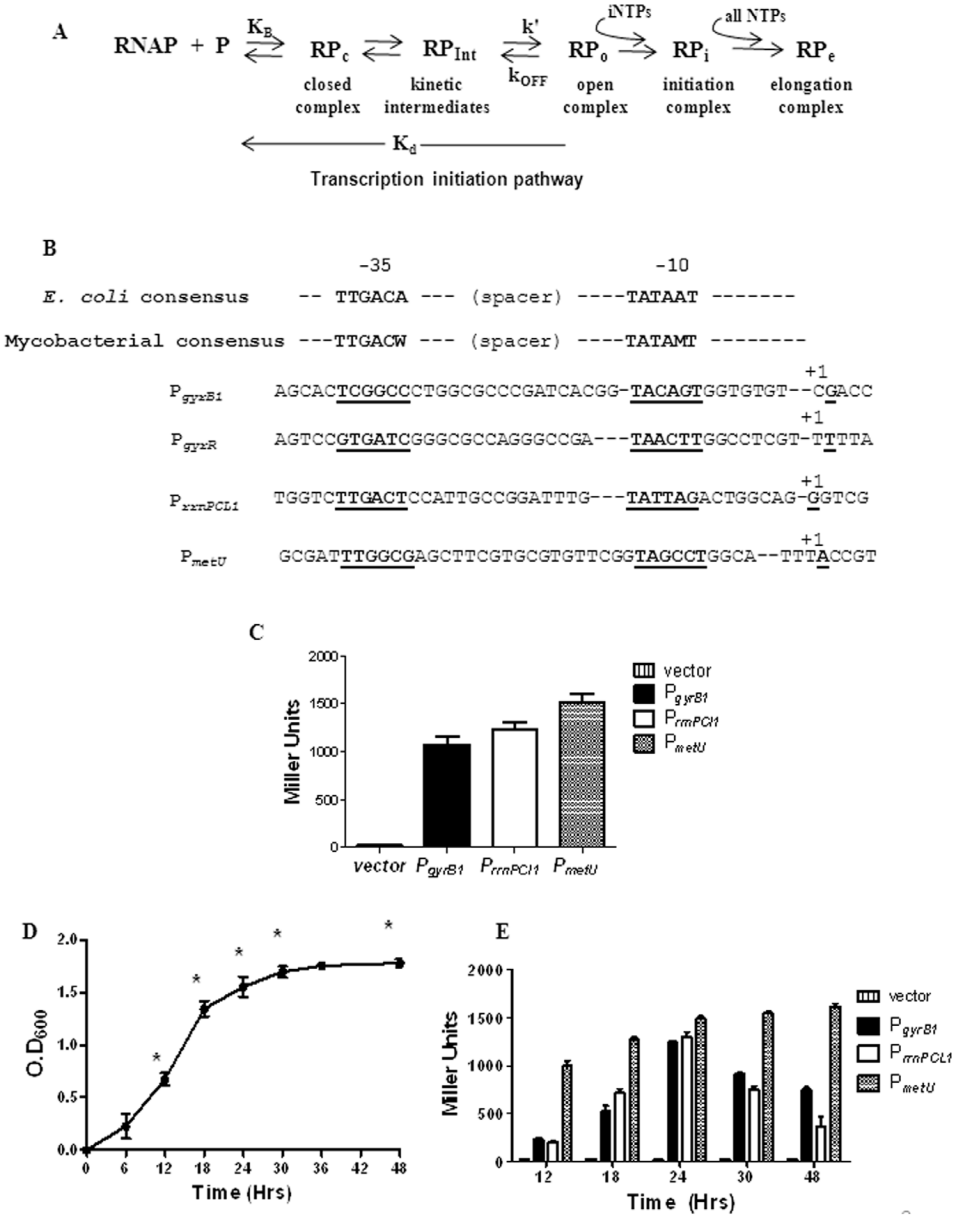
Transcription with *M. tuberculosis* promoters. A Scheme of transcription initiation. B Sequences of promoters used in this study. The -35, -10 elements and the transcription start sites are underlined. Sequences are aligned with *E. coli* σ^70^ and mycobacterial σ^A^ dependent promoter consensus. **C** ***in vivo*** **reporter assays.** Transcriptional activities of the promoters were determined by β-galactosidase reporter assays from the early exponential phase cultures of *M. smegmatis*. The promoter activities are represented in Miller units on Y axis. **D** **Growth curve of** ***M. smegmatis*** **.** The OD_600_ of the culture was recorded at 0, 6, 12, 24, 30, 36 and 48 Hrs. The time points at which the β-galactosidase reporter assays were carried out are indicated with asterisks. **E** ***in vivo*** **reporter assays in different phases of growth.** *M. smegmatis* cells harbouring pSD5B promoter constructs were grown upto 48 hours and promoter activity was determined at different times of growth by β-galactosidase assay as mentioned above.

Transcription constitutes the first stage in gene expression and comprises of multiple steps *viz* initiation, elongation and termination (**Figure 1A** **)**. During the initiation, RNA polymerase (RNAP) binds to the promoter, leading to the formation of several intermediates which differ from each other in their kinetic properties [8]–[11]. After initial binding of RNAP to the promoter to form the closed complex, the DNA strands unwind to form a catalytically competent open complex, associated with a series of conformational changes in the enzyme as well as DNA [8]–[11]. Binding of the initial ribonucleotides (iNTPs) to the RNAP results in the formation of ternary complex, poised to enter into the elongation mode [8]–[11]. After the synthesis of abortive transcripts of 2–15 nucleotides in most of the promoters studied, the RNAP leaves the promoter to enter into the elongation phase of transcription [12]. Because of these elaborate orchestrated steps, the initiation pathway is also fine-tuned by a number of regulatory mechanisms to meet the requirements posed by various physiological conditions of the cell [11], [13]. Typically, a few promoters (*rrn*, initiator tRNA) achieve higher promoter strength in exponential phase due to the stabilization of open complex by initiating nucleotides (iNTPs). In contrast, during the stationary phase, increase in the concentration of guanosine tetra/penta phosphate ((p)ppGpp), leads to inhibition of transcription from these promoters [14], [15]. Thus the strength of these promoters varies with the growth phase as they are subjected to growth phase dependent regulation.

The present work is the first detailed kinetic analysis of the events during transcription initiation in *M. tuberculosis*. We have carried out promoter-polymerase interaction studies using a few of the house-keeping promoters to characterize the mechanisms of transcription initiation. The kinetics of RNAP-DNA interactions was measured in different promoter sequence contexts to determine their key rate–limiting steps. Further, the role of iNTPs and pppGpp in regulating transcription initiation was studied. While the *M. tuberculosis* ribosomal RNA promoter exhibited the characteristics seen with *E.coli* and other bacteria, the promoters for the gyrase operon showed unusual and hitherto unknown pattern of transcription initiation. Most significantly, the promoters for the same genes from *M. smegmatis* and *M. tuberculosis* differed markedly in their kinetic properties and response to the effectors.

## Results

### Promoter Characteristics and Activities

For comparison of the promoter-RNAP interactions, two stable RNA promoters *viz*. ribosomal RNA, initiator tRNA (P*_rrnPCL1_*, P*_metU_*) and another house-keeping promoter (P*_gyrB1_* ) were chosen in addition to a weak promoter (P*_gyrR_*) **(** **Figure 1B** **)**. *M. tuberculosis* has only a single operon for rRNA transcription driven by two promoters [16]–[18]. Amongst the two promoters, P*_rrnPCL1_* is the major house-keeping promoter and is stronger than P*_rrnP1_* [16]. Moreover, P*_rrnPCL1_* is found in the genome of every sequenced species of mycobacteria and appears to be conserved across the genus [18]. P*_metU_* is the only promoter driving the transcription of the single initiator tRNA gene in *M. tuberculosis* [19]. The -10 and -35 elements of these two stable RNA promoters resemble the mycobacterial σ^A^ consensus sequence [6]. Two promoters from the *gyr* operon of *M. tuberculosis* included in the study are illustrated in **Figure 1B** [6]. P*_gyrB1_* is the major promoter of the *gyr* operon that directs the high levels of transcription from the *gyrB - gyrA* dicistron [6]. The -10 element of the promoter is similar to the σ^A^ dependent promoter consensus sequence. P*_gyrR_* is an overlapping and divergently organized promoter to P*_gyrB1_* whose activity is appoximately 13 times weaker than P*_gyrB1_* and hence is a representative weak promoter in this study [6].

The relative *in vivo* activities of the promoters subjected to the present analysis were determined using the promoter-*lacZ* transcriptional fusion constructs transformed to *M. smegmatis mc^2^ 155*. P*_metU_* showed the highest activity followed by P*_gyrB1_* and P*_rrnPCL1_* **(** **Figure 1C** **)**. The activities were also measured at different times of growth (**Figure 1D** **)**. The promoter strength of P*_metU_* did not vary significantly at different growth phases, while the activities of both P*_rrnPCL1_* and P*_gyrB1_* decreased as the cells entered the stationary phase (**Figure 1E** **)** suggesting the regulation of these promoters is growth phase dependent. To understand the mechanism of differential regulation of the promoters, equilibrium and kinetic analysis of transcription initiation was carried out.

### Promoter–polymerase Interaction

**Figure 2:**
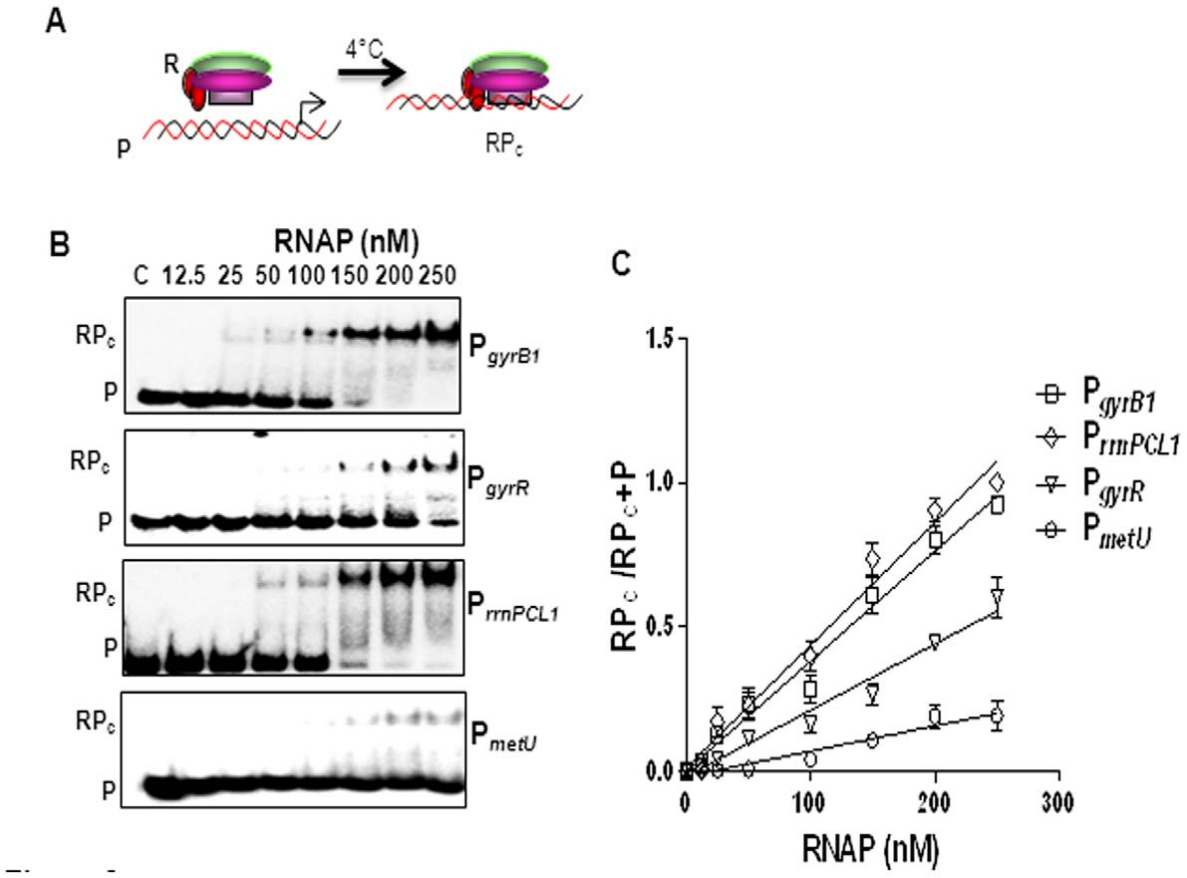
Determination of K_B_. A Scheme of assay, R represents RNAP, P represents promoter fragment and RP_c_ represents closed complex. **B** Promoter fragments were incubated with different RNAP concentrations for 20 min and the complexes formed were resolved using 4% native-PAGE. **C** The amount of radioactivity in bound and free fragments was measured by densitometry and indicated as RP_c_ and P respectively. RP_c_/RP_c_+P ratios were plotted as function of RNAP concentrations. K_B_ was calculated from the slope of the graph. The values obtained are mean of three independent experiments (Table 1).

**Figure 3:**
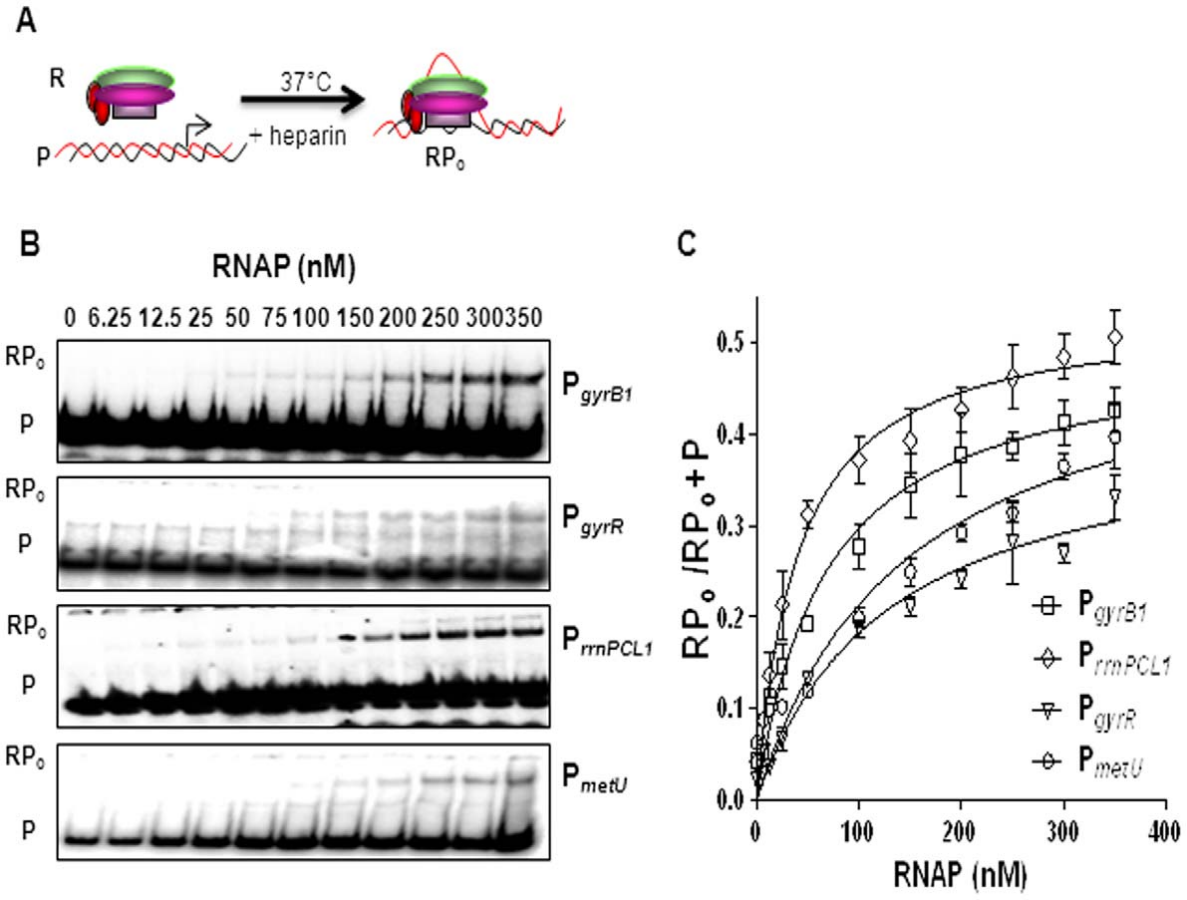
Determination of K_d_. A Scheme of assay, R represents RNAP, P represents promoter fragment and RP_o_ represents open complex. **B** Promoter fragments were titrated using a range of RNAP concentrations, incubated at 37°C for 10 min, challenged with heparin and analyzed using 4% native-PAGE. **C** The amount of radioactivity in bound and free fragments was measured by densitometry and indicated as RP_o_ and P respectively. RP_o_/RP_o_+P ratios were plotted as a function of RNAP concentrations to obtain the hyperbolic graph. The values (Table 1) are mean of three independent experiments.

For the equilibrium binding analysis of *M. tuberculosis* promoters with RNAP, binding constant (K_B_) for closed complex formation was measured. K_B_ is the determinant of the strength of the closed complex, which is obtained by titration of the promoters with the RNAP and plotting the ratios of the bound fraction to total DNA (RP_c_/RP_c_+P) against the RNAP concentrations (**Figure 2A, B** **)**. The affinity of RNAP for P*_rrnPCL1_* and P*_gyrB1_* during closed complex formation was 5 and 3.3 fold higher respectively compared to the other stable RNA promoter, P*_metU_*. K_d_ which is the determinant of the strength of the open complex, was obtained by plotting the ratio of RP_o_/RP_o_+P (fraction DNA bound) against RNAP concentrations (**Figure 3A, B** **,** **Table 1** **)**. From the Table 1 it is apparent that P*_rrnPCL1_* and P*_gyrB1_* have comparable K_d_ values (lower than P*_gyrR_* and P*_metU_*). Thus, although the affinity of RNAP for both P*_rrnPCL1_* and P*_gyrB1_* was higher than other two promoters studied, (see above) the overall extent of open complex formation was low, which could be either due to slow rate of formation or reduced longevity of open complex. Therefore, next,the rates of formation as well as stability of open complexes were measured for all the promoters. The rates of formation of open complex for the promoters were determined by measuring the rate constants of isomerization (see next paragraph). The stability of the open complexes was determined by monitoring the decay of the complexes with time.

**Table 1:** Summary of equilibrium binding constants and kinetic parameters.

Constant/Property	P*_gyrB1_*	P*_rrnPCL1_*	P*_gyrR_*	P*_metU_*
K_B_ (x 10^7 ^M^−1^)	2.1±0.03	3.1±0.16	0.27±.001	0.7±0.08
K_d_ (x 10^7 ^M)	68.05±12.16	40.14±6.09	146.2±44.58	127.6±27.19
k' (fast) (min^−1^)	1.4±0.7	1.2±0.08	0.54±0.20	0.24±0.15
k' (slow) (x 10^−2^ min^−1^)	0.24±0.4	0.21±0.74	0.61±0.24	0.04±0.04
K_B_ x k' (fast)	2.1×10^7^	2.7×10^7^	1.3×10^6^	1.35×10^6^
k_off_ (fast) (min^−1^)	0.74±0.27	2.63±1.41	1.35±0.92	0.48±0.16
k_off_ (slow) (x10^−1^ min^−1^)	0.5±0.17	0.4±0.29	0.63±0.16	0.16±0.09
t_1/2_ (fast) (min)	5.17	1.51	7.75	4.4
t_1/2_ (slow) (min)	17.78	6.12	13.32	34.84
Abortivetranscription	++++	–	+++	–
Promoter clearance (min)	23	2.3	18	8.93

**Figure 4:**
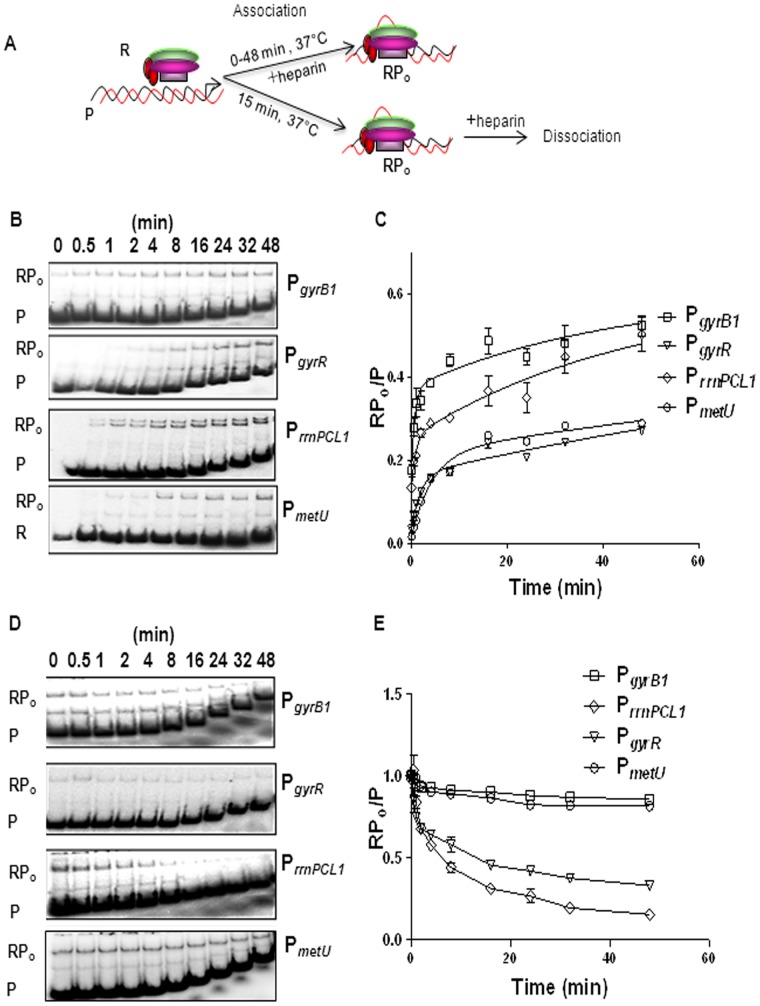
Kinetics of association and dissociation. A Scheme of assay, R represents RNAP, P represents promoter fragment and RP_o_ represents open complex. **B** The association of RNAP and promoter DNA to form open complex was monitored over time intervals ranging from 0 to 48 min, as indicated. **C** The amount of radioactivity in bound and free fragments was measured by densitometry and indicated as RP_o_ and P respectively. RP_o_/P ratios were plotted against time and k′ was measured by double exponential association analysis. The values of k′ are mean of three independent experiments and are shown in Table 1. **D** Open complex at each promoter fragment was formed by incubating RNAP and promoter fragments for 15 min at 37°C. Dissociation of RNAP was monitored by challenging the pre-formed open complex with heparin for time intervals ranging from 0 to 48 min, as indicated. **E** The data was fit into the double exponential decay equation to measure k_off_. The biphasic nature of the double exponential decay curve is suggestive of the existence of two complexes decaying at different rates. The steeper and the trailing parts of the curve represent the faster and slower decaying phases respectively. The values of k_off_ are mean of three independent experiments (Table 1).

Pseudo first order rate constant (k') which is the measure of isomerization rate was calculated by monitoring the time dependent interaction of RNAP and promoters. From the results of association kinetics, (**Figure 4A, B**, **Table 1** **)** it is evident that the rate of open complex formation was highest for the P*_gyrB1_* followed by P*_rrnPCL1_*, thus providing an explaination for the higher strength of these two promoters. Notably, the rate of isomerization at P*_metU_* was slow, although the *in vivo* activity of this promoter was high (see Figure 1 E), suggesting the possibility that the downstream kinetic events after isomerization step could be the key determinants of the high strength of the promoter. Indeed, when the rate of the dissociation of RNAP from the promoter was monitored, RNAP dissociated at a very low rate (**Figure 4C and D** **)**. P*_gyrB1_* also showed slower dissociation rate indicating another contributing feature for its strength. As expected, like other well studied rRNA promoters, higher dissociation rate was seen at P*_rrnPCL1,_* compared to the more stable open complexes at P*_gyrB1_* and P*_metU_* (**Table 1** **)**. Biphasic curve obtained in all these cases could be an indication of an initial faster dissociation of closed complex and a slower dissociation of a comparatively more stable open complex.

### Effect of iNTPs on Open Complex Formation

**Figure 5:**
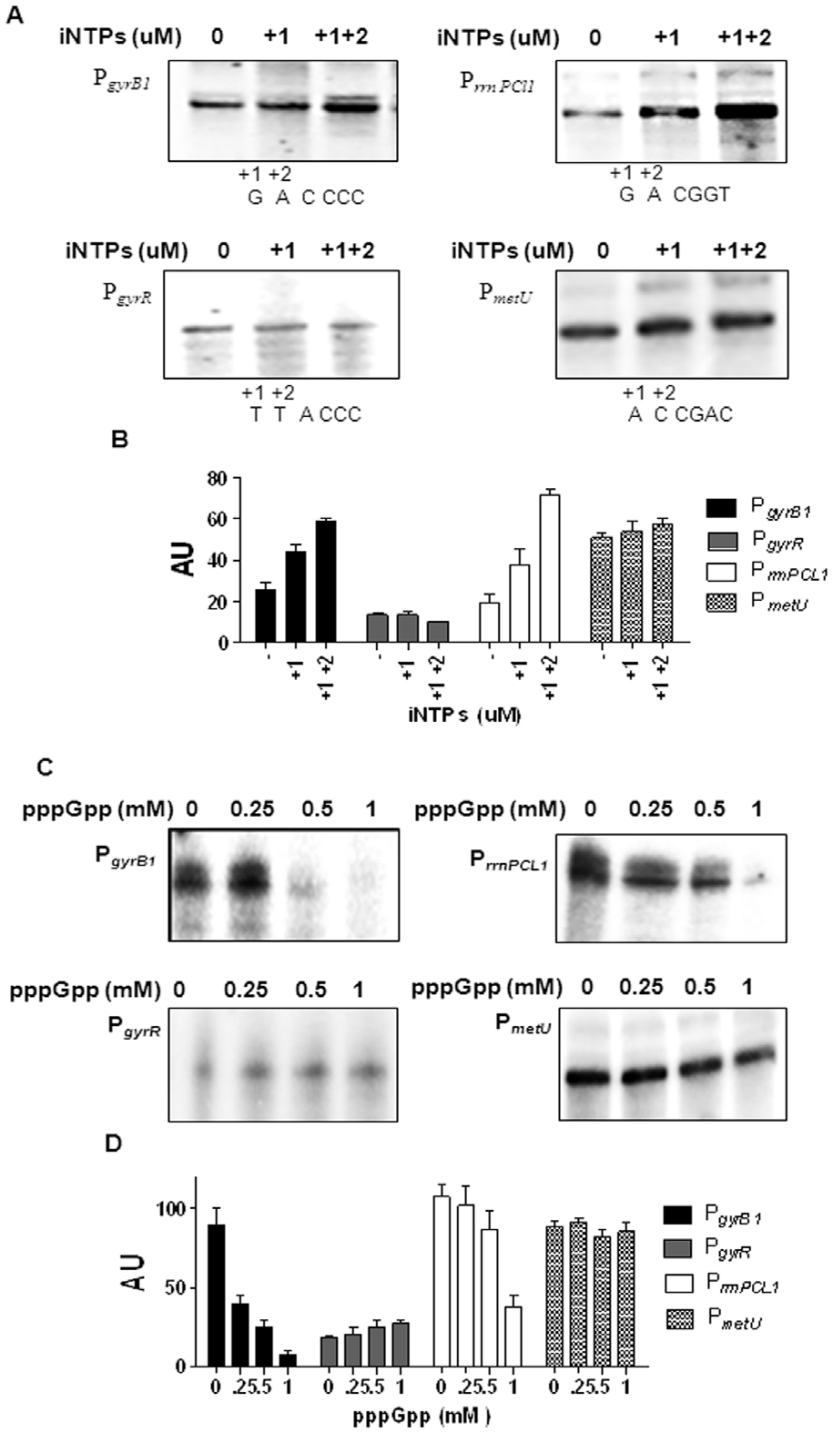
Effect of iNTPs on open complex. A Open complex was formed in the presence of 100 µM of iNTPs (+1 and +1, +2) and the transcription was initiated by adding heparin and all the four NTPs. The run-off transcripts were resolved on 8% urea–PAGE. The initial transcribed sequence (ITS) for all the promoters is shown. **B** The graph shows the quantification of transcripts formed in the absence and presence of +1 iNTP and +1, +2 iNTPs. The amount of run-off transcripts formed was measured by densitometry and indicated as AU on Y axis. **C** **Effect of pppGpp on open complex.** Open complex was formed in the presence of increasing concentrations of pppGpp as indicated and the transcription was initiated as described before. The run-off transcripts were resolved on 8% urea–PAGE. **D** The graph shows the quantification of transcripts formed in the presence of increasing concentrations of pppGpp. The amount of run-off transcripts formed was measured by densitometry and indicated as AU on Y axis.

rRNA promoters from diverse organisms show enhanced promoter activity in the exponential phase. Since the promoter strength of P*_rrnPCL1_* and also P*_gyrB1_* varied with the growth phase (see **Figure 1E** **)**, we next examined the effect of iNTPs on the isomerization and the open complex stability of the P*_rrnPCL1_*, P*_gyrB1_* and the other two promoters. The open complex at P*_rrnPCL1_* increased on addition of +1 iNTP, and in presence of both +1 and +2 iNTPs (**Figure 5A, B** **, Figure S1A, B)**. In addition, the longevity of the open complex at P*_rrnPCL1_* was increased in the presence of the first two NTPs (**not shown)**. In contrast to the rRNA promoter, the other stable RNA promoter, P*_metU_,* did not respond in a similar fashion when the +1 iNTP was added. Notably, the isomerization was stimulated at P*_gyrB1_* in the presence of +1 iNTP with the further increase in the presence of both +1 and +2 iNTPs, similar to the pattern seen with P*_rrnPCL1_* **(** **Figure 5A, B** **, Fig. S1A, B)**. However, unlike P*_rrnPCL1_* the initiating nucleotides did not influence the longevity of the open complex at P*_metU_* and P*_gyrB1_* to a significant extent **(data not shown)**, which could be attributed to the intrinsic stability of the complexes at these promoters in the absence of initiating nucleotides. *In vitro* assays carried out in the presence of pppGpp showed that transcription at P*_rrnPCL1_* and P*_gyrB1_* was inhibited **(** **Figure 5C, D** **)**. There was no significant effect of pppGpp on transcription at P*_metU_* **(** **Figure 5C, D** **),** revealing that the promoter is not subjected to a similar kind of regulation.

### Inefficient Promoter Clearance at Gyr Promoters

**Figure 6:**
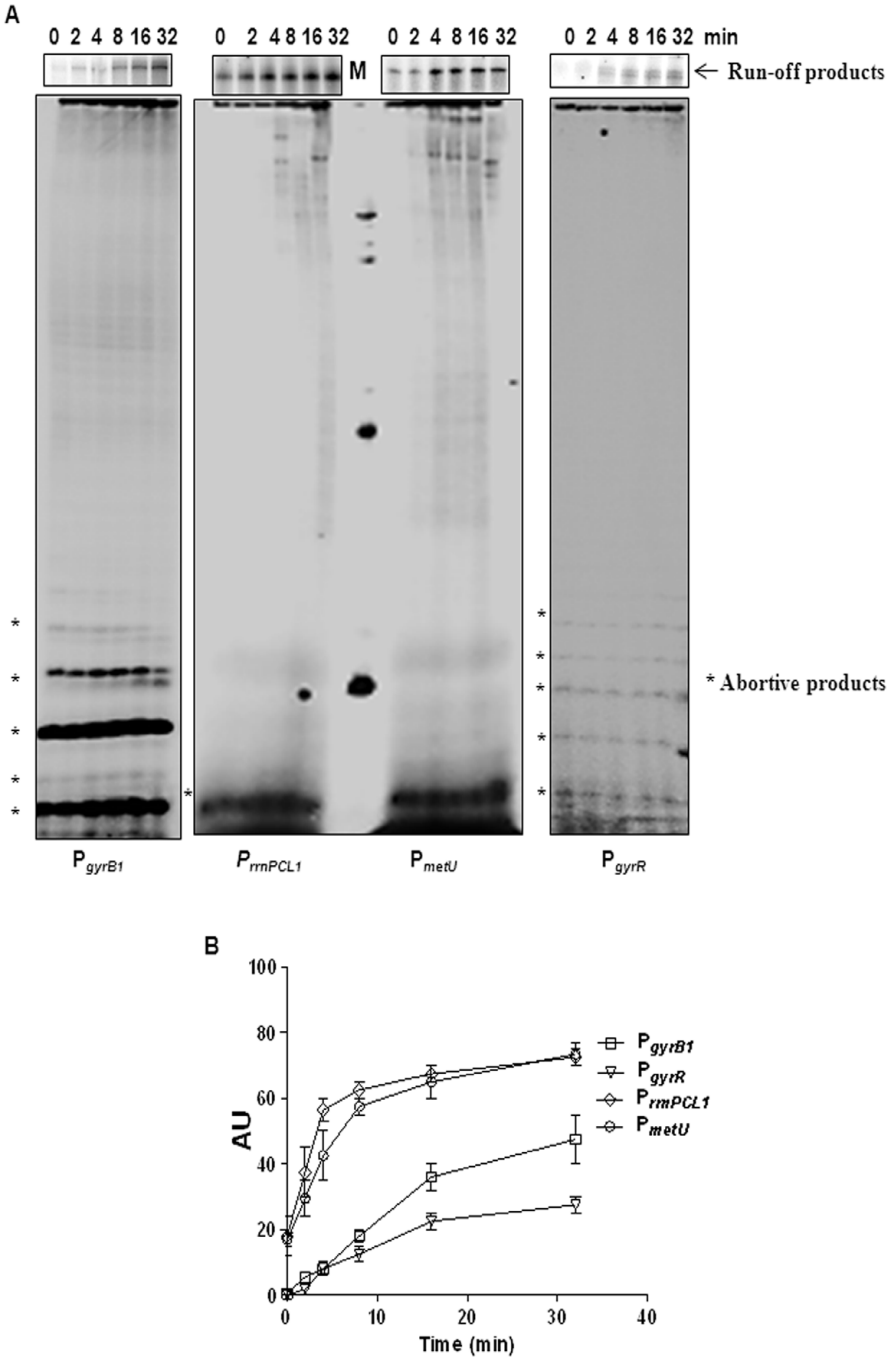
*In vitro* transcription assays. A Promoter clearance assay was carried out as described in Materials and Methods. (α-^32^P) +2 nucleotide of ITS was used to label the transcripts. A 10 µl aliquot from the same assay mix was loaded onto 8% urea–PAGE (19∶1) to resolve the run-off transcripts and onto 23% urea-PAGE (10∶1) to resolve abortive transcripts. Arrow on the right side shows the run-off transcripts, while the asterisks show the abortive transcripts synthesized from the promoters. M on the top of the gel corresponds to marker. The bands from top to bottom in this lane corresponds to 91, 75, 32, and 11 nucleotides. **B** The amount of run-off transcripts was quantified (AU) and plotted against time. The promoter clearance rate was measured from the graph as the time required to form 90% of maximum transcript formed at each promter and is denoted as PC_90%_. The clearance rate for each promoter is the mean of three values (Table 1).

In addition to the efficiency in DNA binding and melting, overall promoter strength also depends on the rate of promoter clearance by RNAP. Thus the extent of abortive initiation during the transition from the initiation to elongation also has an important bearing on transcription initiation [20]. To determine the contribution of the post DNA-melting steps in overall transcription efficiency, the rate of promoter clearance and formation of abortive as well as run-off transcripts were measured **(** **Figure 6A, B** **,** **Table 1** **)**. The rate of promoter clearance was faster at two stable RNA promoters in contrast to the *gyr* operon promoters. The promoter which drives the dicistron transcription (P*_gyrB1_*) had 2.5 and 10 times slower clearance rate compared to P*_metU_* and P*_rrnPCL1_* respectively. The lower clearance rate seen with the *gyr* promoters seems to be resulting out of higher abortive transcription delaying the escape of RNAP from these promoters (**Figure 6** **)**. When the amount of run-off transcripts synthesized at these promoters were compared in the single and multiple round conditions, fewer run-off transcripts were synthesized at P*_metU_* in the single round transcription compared to the P*_gyrB1_* and P*_rrnPCL1_* **(data not shown)**. However, after multiple rounds of transcription, the accumulation of run-off transcripts at P*_metU_* was comparable to that of P*_gyrB1_* and P*_rrnPCL1_*.

## Discussion

Kinetics and equilibrium binding studies provide an insight into the strength and the mechanism of transcription initiation at the promoters. In addition to the sequence of promoter elements and overall promoter architecture, the strength of a given promoter is governed by events occurring at various stages of the transcription initiation process and the *in vivo* strength is the net result of cumulative effect of all the steps. In the present study with the four promoters of *M. tuberculosis*, we have dissected the individual steps in the transcription initiation to understand their characteristic rate limiting steps.

**Figure 7:**
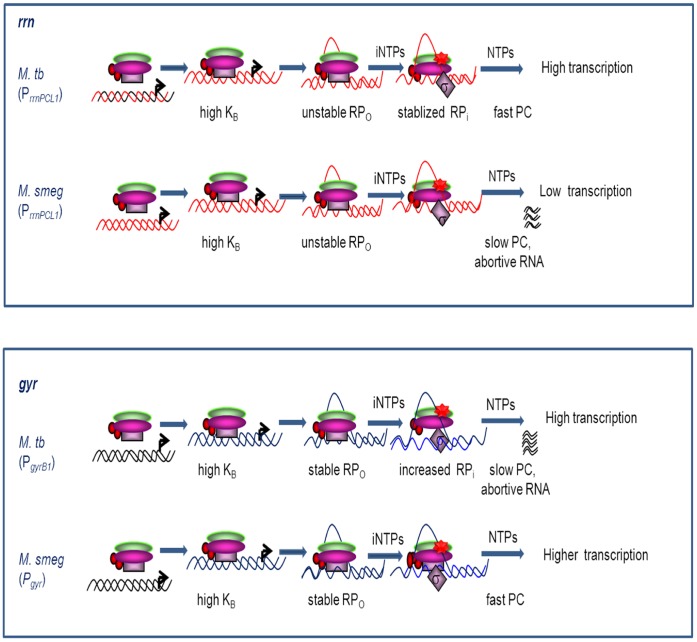
Summary of transcription initiation at *gyr* and *rrn* promoters from *M. tuberculosis* and *M. smegmatis*. Individual equilibrium and kinetic events occurring at *M. tuberculosis* and *M. smegmatis gyr* and *rrn* promoters are compared. RP_c_ and RP_o_ refer to closed complex and open complex respectively, RP_I_ refers to initiation complex, iNTPs refers to initiating nucleotides, PC refers to promoter clearance. iNTPs enhance the formation of RP_I_ at P*_gyrB1_* and stability of open complex at *rrn* promoters.

Generally, in every organism as if by a rule, the *rrn* operons are transcribed by the strongest house-keeping promoters. Very high frequency of initiation at *rrn* promoters is a characteristic feature that contributes to the abundance in rRNA transcripts [21] and the P*_rrnPCL1_* of *M. tuberculosis* is no exception to this paradigm. The high strength of the promoter can be attributed to its −10 and −35 elements, which closely resemble to the σ^A^ consensus sequence [6], [16]. The instability of the open complex and increase in half-life in the presence of iNTPs seen with the promoter is a characteristic property of any typical rRNA promoter analyzed so far including promoters from *M. smegmatis* [15], [22]–[24]. However, the comparison of kinetics at P*_rrnPCL1_* of *M. tuberculosis* with that of *M. smegmatis* also revealed interesting differences (**Figure 7** **)**. P*_rrnPCL1_* of *M. smegmatis* showed slower promoter clearance and greater amount of abortive transcription in addition to an intrinsically unstable open complex [24]. The cumulative effect of these kinetic events results in (10 fold) lower transcriptional activity of *M. smegmatis* P*_rrnPCL1_* in comparison to *M. tuberculosis* (24, **Figure 7** **, Figure S2**). The two promoters also differed significantly in their response to iNTPs and pppGpp (24 and this work); the stimulation and inhibition by the two effectors was much more pronounced at P*_rrnPCL1_* of *M. tuberculosis*. However, inadequacy of the P*_rrnPCL1_* of *M. smegmatis* appears to be compensated by the very strong P*_rrnB_*_,_ which appears to be one of the strongest promoter in the organism. Moreover, the presence of a second functional rRNA operon also ensures adequate rRNA transcription. All these observations indicate the importance of species specific variations in promoters to meet the cellular requirements. The constitutive high level transcripts synthesized from the single rRNA operon of *M. tuberculosis* seem to fulfill the need of the metabolic machinery of the cell possibly due to the slow growth characteristics of the organism. As a consequence, the present day *M. tuberculosis* strains and other closely related pathogens seem to have lost the second rRNA operon (*rrnB*) found in the fast growing species of the genus [16], [17]. The down-regulation of the operon by the action of the pppGpp during transcription initiation would ensure the fine tuning of rRNA expression to lower levels sufficient in the stationary phase. One would expect a very low level of rRNA expression in the dormant state of the organism during its intracellular survival. The positive regulation of the P*_rrnPCL1_* by iNTPs and inhibition of its transcription initiation by pppGpp point out at the remarkable conservation of rRNA transcription regulation across the diverse bacterial species and this sensing mechanism appears to provide a unified theme for the growth phase dependent regulation of rRNA transcription. However, as summarized in Table S1, the rRNA operons are subjected to diverse controls, in addition to conserved features, adding another level of complexity.

Surprisingly, in contrast to the *rrn* promoter, the other stable RNA promoter, P*_metU,_* which drives the transcription from a single initiator tRNA gene in *M. tuberculosis,* does not appear to be influenced by iNTPs and pppGpp. In this context, studies with *E.coli* tRNA promoters provide interesting parallels. *E. coli* has two initiator tRNA promoters, P*_metY_* and P*_metZ_,* transcribing the single initiator tRNA and an operon containing three tandemly repeated tRNA_f1_^Met^ respectively [25]. However, they respond differently to these regulators**.** While the transcription from P*_metY_* is unaffected by pppGpp, the P*_metZ_* is subjected to inhibition [26]. Thus, it is apparent that unlike the *rrn* operons, regulation of promoters of initiator tRNA is not universally conserved. Also in contrast to the P*_rrnPCL1_*, the isomerization step was found to be the rate limiting step at P*_metU_* in *M. tuberculosis*. The lower amounts of run-off transcripts from the promoter in single round transcription assays, compared to the *gyr* and *rrn* promoters could be due to the slower rate of formation of the open complex. However, the high promoter strength of the P*_metU_* under *in vivo* conditions could be accounted by its higher open complex stability, faster promoter clearance and low levels of abortive transcription. The accumulation of large amounts of transcripts after multiple rounds of transcription at P*_metU_* (**Figure 4D**) could be explained not only because of its promoter strength but also possibly due to frequent recycling of RNAP likely to occur at the tRNA genes. The process of re-initiation of transcription could be facilitated because of the presence of an intrinsic terminator at the end of the short gene to allow RNAP to fall off at a distance not far away from the promoter [19], [27]. Notably, high levels of transcripts seen with class III transcripts in eukaryotes is attributed to efficient recycling of the Pol III on tRNA genes [28].

The extent of closed complex formation and rate of isomerization are the major determinants of promoter strength of P*_gyrB1_*, the major promoter transcribing the *gyr* operon of the organism. The rate limiting step at P*_gyrB1_* seems to be the promoter clearance by RNAP. The promoters efficient at early steps of promoter-polymerase interaction tend to be inefficient in promoter clearance [29]. The present data with P*_gyrB1_* supports this hypothesis. The stimulation of open complex formation upon addition of the iNTPs and the opposing effect of inhibition by pppGpp is an unusual property of the promoter, a feature distinct from P*_gyr_* of *M. smegmatis,* which do not appear to respond in a similar fashion [24]. To our knowledge, this is the first ever description of nucleotide mediated activation and pppGpp mediated inhibition of transcription initiation of *gyr* promoter in any organism or for that matter promoter of any topoisomerase gene.

Surprisingly, the present studies reveal that the process of transcription initiation at *M. tuberculosis* P*_gyrB1_* is markedly distinct from the P*_gyr_*, a single promoter transcribing gyrase operon of *M. smegmatis*, which is a non- pathogenic member of the same genus often used as surrogate host for a variety of studies (**Table S2**). Slower isomerization rates, faster promoter clearance, lower abortive initiation are the characteristic features of the transcription initiation at P*_gyr_* from *M. smegmatis* [24]. In contrast, the principle *gyr* promoter of *M. tuberculosis*, the subject of the present analysis, exhibited entirely opposite effects *viz*. faster rate of open complex formation, slower promoter clearance and higher abortive transcription. Notably, the two promoters exhibit markedly distinct mode of ‘Relaxation Stimulated Transcription’ (RST), a homeostatic control employed by cells to regulate gyrase activity and topological status of the genome [6], [30], [31]. In addition, distinct influence exerted by iNTPs and pppGpp on the promoter strength of P*_gyrB1_* could ensure control of the promoter linked to the growth phase. These differences in the regulation of the *gyr* operons between the two different species may indeed reflect their growth rates, physiology and contrastingly different life-style.

To conclude, during initiation of transcription, each of the *M. tuberculosis* promoters studied is subjected to different rate-limiting steps and regulation. While unstable open complexes appear to serve as the sensors of initiating nucleotide concentration in rRNA promoter, distinctly, tRNA promoter is rate-limited at open complex formation and not subjected to growth phase dependent control. The opposing effects of the regulatory effectors, on the principle promoter of the *gyr* operon of the organism indicate the fine control connecting growth phase to supercoiling homeostasis of the genome, a mechanism probably required for metabolic shut down.

## Materials and Methods

### Promoter DNA, Transcription Templates and RNAP

The strains, plasmids and the sequences of the promoter fragments used for this study are listed in the **Table S3**. Since promoters of *gyr* operon are divergent and overlap, the sequences of these promoters were altered such that only one of the two promoters was functional. The sequence of -10 element of P*_gyrB1_* was rearranged from TACAGT to ACTTAG in the fragment containing P*_gyrR_* and the sequence corresponding to -10 element of P*_gyrR_* was changed from TCTTCT to CTCGTG in fragment containing P*_gyrB1_* (**Figure 1B**).

pARN104, a derivative of pUC18 was used as a vector to clone the promoter fragments amplified from *M. tuberculosis H37Ra* genomic DNA with specific primers. For *in vitro* transcription assays, templates were prepared by PCR amplification from the constructs using a set of vector specific primers followed by gel purification. The primers used in this study are listed in Table S4. RNAP was isolated from *M. smegmatis* SM07 [32] by a modified procedure involving *in vivo* reconstitution of the enzyme with σ^A^ [33]. The σ^A^ content in the RNAP preparation was 95% stoichiometric to the β, β' subunits. The specific activity of the purified RNAP was determined both by the standard method of ^3^[H]-UTP incorporation and by titrating the promoter fragment with a range of RNAP concentrations as described [34], [35]. pppGpp was synthesized as described [36].

### β-galactosidase Reporter Assays

The cells were grown in MB7H9 (Difco) medium supplemented with 2% glucose (Sigma) and 0.05% Tween80 (Sigma). Promoter strength was measured by β-galactosidase reporter assay and the activity represented in Miller units (Miller units = 1,000×*A*_420/_(time (min) x volume of culture (ml) x optical density at 600 nm] [37]. *M*. *smegmatis mc^2^155* transformed with the vector pSD5B [38] was used as the negative control. To determine the *in vivo* promoter strength in different growth phases, the cultures of *M*. *smegmatis mc^2^155* transformed with the promoter fusion constructs were grown for 12, 18, 24 30, 48 hours and the β-galactosidase reporter assay was carried out as described before.

### Electrophoretic Mobility Shift Assay (EMSA)

For EMSA, oligonucleotides having the individual promoter sequences were used. The 5' promoter fragments were end labeled at their 5' ends of one of the strands with (γ -^32^P) ATP and T4 polynucleotide kinase (New England Biolabs) at 37°C for 30 min. The labeled strand was annealed with two molar excess of complementary strand. The binding reactions were carried out in transcription buffer containing 50 mM Tris HCl, (pH-8.0 at 25°C), 3 mM magnesium acetate, 100 µM EDTA, 100 µM DTT, 50 mM KCl, 50 µg ml^−1^ BSA, 5% glycerol [27]. The buffer used for pppGpp assays also included 35 mM of potassium glutamate. The electrophoresis was carried out either at 4°C or room temperature on a 4% native-PAGE. The amount of radioactivity in bound and free promoter fragments was measured by phosphorimager (Fujifilm) and densitometry analysis by Image Guage ver. 2.54.

### Determination of Equilibrium Constants (K_B_ and K_d_)

To study the RNAP-DNA closed complexes, 1 nM of promoter fragments were titrated with varied amounts of RNAP. The incubation was carried out at ice for 20 min and the fractions were resolved on 4% native-PAGE at 4°C. The K_B_ was determined by Prism software from three independent sets of experiments as described [39]. For determination of the K_d_ of the open complex, different concentrations of RNAP and 1 nM of promoters were incubated at 37°C for 10 min followed by heparin (50 µg ml^−1^) challenge for 1 min.The fractions were resolved on 4% native-PAGE at 37°C. The equilibrium dissociation constant for the heparin resistant complexes (K_d_) was measured by the equation Y = Y_max_[RNAP]/K_d_+[RNAP], where Y_max_ corresponds to binding maximum [40], [41].

### Determination of Association and Dissociation Rate Constants

For determination of association rate constants, closed complexes were pre-formed as described. The aliquots (9 µl) from the assay mixture were withdrawn at different time points (0 to 48 min) and challenged with heparin (50 µg ml^−1^) followed by immediate loading onto 4% native-PAGE electrophoresed at room temperature to analyze the bound fractions. For dissociation assays, open complexes were formed by incubating promoter fragments and RNAP for 15 min at 37°C and the assay mixtures were subjected to heparin challenge (50 µg ml^−1^). Aliquots (10 µl) were withdrawn at time intervals ranging from 0 to 48 min followed by loading onto 4% running native-PAGE electrophoresed at room temperature. The first order and dissociation rate constants were calculated by fitting the values as described earlier [34].

### Assays to Determine the Effect of Ribonucleotides on Isomerization and Stability

Initially promoter DNA was incubated with RNAP (50 nM; 100 nM in case of P*_gyrR_*) in the presence of ribonucleotides. The ribonucleotides were added to a final concentration of 100 µM in different combinations (+1, +1+2). The reactions were incubated to form competitor resistant complex as described above and supplemented with NTP mix (100 µM), 1 µCi (α- P^32^) UTP and incubated at 37°C for 15 min. The reactions were terminated with 2x stop dye (95% formamide, 0.025% (w/v) bromophenol blue, 0.025% (w/v) xylene cyanol, 5 mM EDTA and 0.025% SDS and 8 M urea). The samples were kept at 95°C for 1 min and snap chilled before loading onto 8% urea-PAGE. The effect of iNTPs on open complex was also checked by EMSA. Briefly, the labeled promoter fragments were incubated with RNAP (50 nM; 100 nM in case of P*_gyrR_*) in the presence of ribonucleotides (100 µM). The reactions were incubated to form competitor resistant complex as described above.

### *In vitro* Transcription Reactions

After RNAP [100 nM] and promoter DNA (50 nM) were incubated at 37°C for 10 min for open complex formation, RNA synthesis was initiated by the addition of NTP mix (100 µM), 1 µCi (α- P^32^) UTP and incubated at 37°C for 15 min and terminated with 2x stop dye (95% formamide, 0.025% (w/v) bromophenol blue, 0.025% (w/v) xylene cyanol, 5 mM EDTA and 0.025% SDS and 8 M urea). The samples were heated at 95°C for 1 min and snap chilled before loading onto 8% urea-PAGE. For single round transcription, 50 µg ml^−1^ heparin was added along with NTP mix (100 µM) and 1 µCi (α- P^32^) UTP. For promoter clearance analysis, promoter DNA (50 nM) and RNAP (100 nM) were incubated in transcription buffer and the reactions were carried out as described (13, 32). +2 NTP in the Initial Transcribed Sequence (ITS) of each promoter was used as the labeled nucleotide. (α- P^32^) ATP was used to label the transcripts in case of P*_gyrB1_*_,_ P*_metU_* (α- P^32^) UTP for P*_rrnPCl1_* and P*_gyrR_*. The samples were analyzed in 23% urea-PAGE (10∶1) to resolve abortive transcripts. A 10 µl aliquot from the same assay mix was loaded onto 8% urea – PAGE (19∶1) to resolve the run-off transcripts. The run-off transcripts were quantified as arbitrary units (AU) and plotted against time. The time corresponding to 90% of the maximum transcript formed, at each promoter, was calculated as the promoter clearance rate (PC_90%_).

### Assays with pppGpp

For the assays with pppGpp, RNAP (100 nM) was incubated with pppGpp (1 mM) in transcription buffer (with 35 mM potassium glutamate) for 15 min. *in vitro* transcription assays to study the effect of pppGpp were carried out as described above.

## Supporting Information

Figure S1 **Effect of iNTPs on formation and dissociation of open complex. A** iNTPs were incubated with promoter fragments and RNAP as described in Materials and Methods to determine their effect on isomerization. The initial transcribed sequence of each template is shown on the left side of the picture. **B** The amount of RNAP-promoter complex formed in the presence and absence of iNTPs was quantified (AU) and plotted. Slower moving complex was quantified in case of P*_rrnPCL1_*.
(TIF)

Figure S2 ***Iin vivo*** **promoter activity of P** ***_rrnPCL1_*** **from** ***M.smegmatis*** **and** ***M.tuberculosis*** **.** *in vivo* activites of P*_rrnPCL1_* from *M. smegmatis* (*M.smeg*) and *M. tuberculosis* (*M.tb*) was measured by β galactosidase assay and plotted on Y axis as Miller units.
(TIF)

Table S1 **Comparison of transcription at** ***rRNA*** **promoter** ***s*** **of** ***E. coli, M. smegmatis*** **and** ***M. tuberculosis*** **.**
(PDF)

Table S2 **Comparison of transcription at** ***gyr*** **promoter** ***s*** **of** ***E. coli, M. smegmatis*** **and** ***M. tuberculosis*** **.**
(PDF)

Table S3 **Strains, plasmids and oligonucleotides used in this study.**
(PDF)

Table S4 **Sequence of primers used in this study.**
(PDF)
