# Supplementary material for: Distinct and Contrasting Transcription Initiation Patterns at Mycobacterium tuberculosis Promoters
Source: PLoS One. 2012 Sep 7;7(9):e43900. doi: 10.1371/journal.pone.0043900 (PMC3436766; doi:10.1371/journal.pone.0043900)
Supplement: Table S1 — Comparison of transcription at rRNA promoter s of E. coli, M. smegmatis and M. tuberculosis . (PDF) [file pone.0043900.s003.pdf]

**Table S1** Comparison of transcription at *rRNA* promoters of *E. coli*, *M. smegmatis* and *M. tuberculosis*

| | *E. coli* | *M. smegmatis* | *M. tuberculosis* | Reference |
|---|---|---|---|---|
| Operons | 7<br>(*rrnA* to *rrnH*) | 2<br>(*rrnA* and *rrnB*) | 1<br>(*rrnA*) | 1-3 |
| Promoters | *rrnB* P1, P2 | *rrnB* –P1, P2<br>*rrnA*- P1, P2 , PCL1 | P1, PCL1 or P3 | |
| Rate limiting step | $RP_o$ stability | $RP_o$ stability–($P_{rrnB}$ )<br>Promoter clearance-($P_{rrnPCL1}$) | $RP_o$ stability-($P_{rrnPCL1}$) | 3-5, This work |
| Growth Phase Dependent Control | ↑iNTP<br>P1<br>↓ppGpp | ↑iNTP<br>$P_{rrnB}$<br>↓pppGpp | ↑iNTP,<br>$P_{rrnPCL1}$<br>↓ pppGpp | |
| UP elements | Stimulate transcription | absent | absent | 1,6 |
| Regulators | FIS, HNS | FIS absent | FIS absent | 1,7 |
