# Supplementary material for: Distinct and Contrasting Transcription Initiation Patterns at Mycobacterium tuberculosis Promoters
Source: PLoS One. 2012 Sep 7;7(9):e43900. doi: 10.1371/journal.pone.0043900 (PMC3436766; doi:10.1371/journal.pone.0043900)
Supplement: Table S2 — Comparison of transcription at gyr promoter s of E. coli, M. smegmatis and M. tuberculosis . (PDF) [file pone.0043900.s004.pdf]

Table S2 Comparison of transcription at *gyr* promoters of *E. coli*, *M. smegmatis and M. tuberculosis*

	E. coli	*M. smegmatis*	*M. tuberculosis*	Reference
Gene organization	Distant loci; *gyr*A -50.32 min, *gyr*B- 83.5min	Dicistronic operon	Dicistronic operon	8-10
Promoters	Extended -10 - (*gyr*A) Canonical (-10,-35) - (*gyr*B)	P_{gyr} - single promoter	P_{gyrB1}, P_{gyrR} - overlapping divergent	
Rate limiting step	Promoter clearance	RP_o formation	Promoter clearance	5,11, This work
Growth Phase Dependent Control	*gyr* A only	ND[ψ]	↑iNTPs ↓ pppGpp	
RST[Φ]	Promoter proximal elements	CHPS* , promoter distal elements	Overlapping promoters	9,10,12

* Cruciform Hairpin Palindromic Sequence
Φ Relaxation Stimulated Transcription
ψ Not detectable
