# Supplementary material for: Distinct and Contrasting Transcription Initiation Patterns at Mycobacterium tuberculosis Promoters
Source: PLoS One. 2012 Sep 7;7(9):e43900. doi: 10.1371/journal.pone.0043900 (PMC3436766; doi:10.1371/journal.pone.0043900)
Supplement: Table S3 — Strains, plasmids and oligonucleotides used in this study. (PDF) [file pone.0043900.s005.pdf]

Table S3 Strains, plasmids and oligonucleotides used in this study

Name	Description	Reference
M.Smegmatis SM07	(HygR, the rpoC of the genome is replaced with the rpoC with a hexa-histidine coding tag at 3' end.)	13
M.tuberculosis H37Ra	(A non-virlulent laboratory strain of *M .tuberculosis*)	
E.coli DH10B	Δ(mrr-hsd RMS-mcrBC) mcrA recA1 Laboratory stock	
pARN104	A derivative of pUC18	This work
pSD5B	Shuttle vector for genetic manipulation and molecular analysis of mycobacteria	14
pET21b- relNTD	pET21b inserted with rel-NTD	15
P_{gyrB1}sense	GGTAAAAACGAGGCCGAGCACTCGGCCCTGGC GCCCGATCACGGTACAGTGGTGTGCGACCCCCT GCGGCGACTC	This work
P_{gyrB1} antisense	CCATTTTTGCTCCGGCTCGTGAGCCGGGACCGC GGGCTAGTGCCATGTCACCACACGCTGGGGGAC GCCGCTGAG	This work
P_{gyrR} sense	AGATGGGTAAAAACGAGGCCAGAAGATCGGCC CTGGCGCCCGATCACGGACTTAGGGTGTGCGAC CCCCTGCGGC	This work
P_{gyrR} antisense	TCTACCCATTTTTTGCTCCGTCTTCTAGCCGGGA CCGCGGGCTAGTGCCTGAATCCCACACGCTGG GGGACGCCG	This work
$P_{rrnPCL1}$ sense	TCACCTATGGATATCTATGGATGACCGAACCTG GTCTTGACTCCATTGCCGGATTTGTATTAGACTG GCAGGGTCGCCCCGAAGCGGGCGG	This work
$P_{rrnPCL1}$ antisense	AGCGGGGTGGATACCTATAGATACCTACTGGCT TGGACCAGAACTGAGGTAACGGCCTAAACATAA TCTGACCGTCCC GCTTCGCCCGCC	This work
P_{metU} sense	GCATGAGGCCGGGGCGCTAAACCGCTCGAAGC ACGCACAAGCCATCGGACCGTAAATGGCTGCGC CCCACCTCG	This work
P_{metU} antisense	CGTACTCCGGCCCCCGCGATTTGGCGAGCTTCGT GCGTGTTCGGTAGCCTGGCATTTACCGACGCGG GGTGGAGC	This work
