# Supplementary material for: Distinct and Contrasting Transcription Initiation Patterns at Mycobacterium tuberculosis Promoters
Source: PLoS One. 2012 Sep 7;7(9):e43900. doi: 10.1371/journal.pone.0043900 (PMC3436766; doi:10.1371/journal.pone.0043900)
Supplement: Table S4 — Sequence of primers used in this study. (PDF) [file pone.0043900.s006.pdf]

Table S4 Sequence of primers used in this study

Name	Description
P_{gyrB1} Forward	CCGAGCTCTCGGCCCTGGCGCCCGACCACGG
P_{gyrB1} Reverse	CCGGAGCTCGCCTCGTCGACCGCGTTGTC
P_{gyrR} Forward	CCGAGCTCTCGGCCCTGGCGCCCGACCACGG
P_{gyrR} Reverse	ACGAGCTCGTGATCGGGCGCCAGGGCCG
$P_{rrnPCL1}$ Forward	TGGAGCTCGACGGTCACCTATGGATATCTATG
$P_{rrnPCL1}$ Reverse	AAGAGCTCTCCAAACAAAACCCAAACACTCCC
P_{metU} Forward	AAGAGCTCCGCTGATCTGGGCGGTCGTAC
P_{metU} Reverse	CTGAGCTCCGAAGCTTGACGTTCAGACCG

Supplementary references

1. Gourse RL, Gaal T, Bartlett MS, Appleman JA, Ross W (1996) rRNA transcription and growth rate-dependent regulation of ribosome synthesis in *Escherichia coli*. Annu Rev Microbiol 50: 645-677.
2. Gonzalez-y-Merchand JA, Colston MJ, Cox RA (1996) The rRNA operons of *Mycobacterium smegmatis* and *Mycobacterium tuberculosis*: comparison of promoter elements and of neighboring upstream genes. Microbiology 142 (Pt 3): 667-674.
3. Verma A, Sampla AK, Tyagi JS (1999) *Mycobacterium tuberculosis rrn* promoters: differential usage and growth rate-dependent control. J Bacteriol 181: 4326-4333.
4. Gaal T, Bartlett MS, Ross W, Turnbough CL, Jr Gourse RL (1997) Transcription regulation by initiating NTP concentration: rRNA synthesis in bacteria. Science 278: 2092-2097.
5. China A, Tare P, Nagaraja V (2010) Comparison of promoter-specific events during transcription initiation in mycobacteria. Microbiology 156: 1942-1952.
6. Arnvig KB, Gopal B, Papavinasasundaram KG, Cox RA, Colston MJ (2005) The mechanism of upstream activation in the *rrnB* operon of *Mycobacterium smegmatis* is different from the *Escherichia coli* paradigm. Microbiology 151: 467-473.
7. Cole ST, Brosch R, Parkhill J, Garnier T, Churcher C, et al. (1998) Deciphering the biology of *Mycobacterium tuberculosis* from the complete genome sequence. Nature 393: 537-544.
8. Bachmann, BJ (1987) Linkage map *of Escherichia coli* K-12,edition 7. In *Escherichia Coli* and *Salmonella Typhimurium* (ed F.C. Neidhardt). 807–877. Washington: American Society for Microbiology.
9.Unniraman S, Nagaraja V (1999) Regulation of DNA gyrase operon in *Mycobacterium smegmatis*: a distinct mechanism of relaxation stimulated transcription. Genes to Cells 4: 697–706.
10.Unniraman S, Chatterji M, Nagaraja V (2002) DNA gyrase genes in *Mycobacterium tuberculosis*: a single operon driven by multiple promoters. J Bacteriol 184: 5449-5456.
11. Menzel R, Gellert M (1983) Regulation of the genes for *E.coli* DNA gyrase: homeostatic control of DNA supercoiling. Cell 34: 105–113.
12. Straney R, Krah R, Menzel R (1994) Mutations in the -10 TATAAT sequence of the gyrA promoter affect both promoter strength and sensitivity to DNA supercoiling. J Bacteriol 176: 5999–6006.
13. Mukherjee R, Chatterji D (2008) Stationary phase induced alterations in mycobacterial RNA polymerase assembly: A cue to its phenotypic resistance towards rifampicin. Biochem Biophys Res Commun 369: 899-904.
14. Jain S, Kaushal D, DasGupta SK, Tyagi AK (1997) Construction of shuttle vectors for genetic manipulation and molecular analysis of mycobacteria. Gene 190: 37-44.
15. Jain V, Saleem-Batcha R, China A, Chatterji D (2006) Molecular dissection of the mycobacterial stringent response protein Rel. Protein Sci 15: 1449-1464.
